# Virulence factors in carbapenem-resistant hypervirulent *Klebsiella pneumoniae*

**DOI:** 10.3389/fmicb.2023.1325077

**Published:** 2023-11-30

**Authors:** Gabriel Mendes, Maria Leonor Santos, João F. Ramalho, Aida Duarte, Cátia Caneiras

**Affiliations:** ^1^Microbiology Research Laboratory on Environmental Health, Institute of Environmental Health (ISAMB), Associate Laboratory TERRA, Faculty of Medicine, Universidade de Lisboa, Lisbon, Portugal; ^2^Faculty of Pharmacy, Universidade de Lisboa, Lisbon, Portugal; ^3^Egas Moniz Center for Interdisciplinary Research, Egas Moniz School of Health and Science, Almada, Portugal; ^4^Institute of Preventive Medicine and Public Health, Faculty of Medicine, Universidade de Lisboa, Lisbon, Portugal

**Keywords:** *Klebsiella pneumoniae*, siderophores, high-risk clones, ST11, ST23, ST13, KPC-variants, treatment

## Abstract

Hypervirulence and carbapenem-resistant have emerged as two distinct evolutionary pathotypes of *Klebsiella pneumoniae*, with both reaching their epidemic success and posing a great threat to public health. However, as the boundaries separating these two pathotypes fade, we assist a worrisome convergence in certain high-risk clones, causing hospital outbreaks and challenging every therapeutic option available. To better understand the basic biology of these pathogens, this review aimed to describe the virulence factors and their distribution worldwide among carbapenem-resistant highly virulent or hypervirulent *K. pneumoniae* strains, as well as to understand the interplay of these virulence strains with the carbapenemase produced and the sequence type of such strains. As we witness a shift in healthcare settings where carbapenem-resistant highly virulent or hypervirulent *K. pneumoniae* are beginning to emerge and replace classical *K. pneumoniae* strains, a better understanding of these strains is urgently needed for immediate and appropriate response.

## 1 Introduction

*Klebsiella pneumoniae*, first described by Carl Friedlander in 1882, is a capsulated, anaerobic facultative, and rod-shaped gram-negative bacterium. This pathogen is found ubiquitously in nature, including plants, soil, animals, and medical devices (Bagley, 1985). In humans, *K. pneumoniae* strains colonize the mucosal surfaces, including the gastrointestinal tract and oropharynx and from these sites, they can gain access to other tissues and cause life-threatening infections. Indeed, *K. pneumoniae* is a highly opportunistic and resilient bacterium responsible for a wide range of infections, including pneumonia, urinary tract infections, bacteremia, and liver abscesses (Paczosa and Mecsas, 2016).

Historically, classical strains of *K. pneumoniae* strains caused severe infections mainly in immunocompromised (usually hospitalized) individuals, which were usually treated with β-lactams (Martin and Bachman, 2018). However, over the past few decades, there has been a worrying amount of acquisition of resistance genes in classical *K. pneumoniae* strains. Consequently, high morbidity and mortality rates have been reported worldwide, mainly due to the lack of effective therapeutic options, resulting in a rising public health threat (Algammal et al., 2023). Additionally, this exponential increase in antimicrobial resistance has had a massive economic impact, mainly related to prolonged hospital stays and high costs for the development and research of new antibiotics (Arato et al., 2021). Importantly, in the *Enterobacteriaceae* family, *K. pneumoniae* strains that acquire resistance to carbapenems belong to the carbapenem-resistant *Enterobacteriaceae* (CRE), which are considered a ‘critical concern’ according to the World Health Organization (WHO, 2017).

In contrast, over the past two decades, “hypervirulent” *K. pneumoniae* (hvKp) strains have gained worldwide attention as a clinically significant pathogen capable of causing severe and invasive infections (mainly community-acquired) in otherwise healthy individuals. Furthermore, although most hvKp strains are susceptible to antibiotics, except for ampicillin, the mortality rate of hvKp infections is high, ranging from 3 to 42% (Lee et al., 2017).

For a long time, the multidrug-resistant (MDR) and hypervirulent pathotypes of *K. pneumoniae* were thought not to overlap, since the MDR pathotype was usually exhibited by classical *K. pneumoniae* strains while hvKp rarely exhibited high levels of antibiotic resistance. Furthermore, the two pathotypes appear to occupy distinct clonal lineages (Bialek-Davenet et al., 2014; Wyres et al., 2019). However, several reports of MDR-hvKp have emerged in recent years (Tang et al., 2020).

Because of this convergence, the number of severe infections in both immunocompromised and healthy individuals has increased due to the lack of appropriate therapeutic options, representing a new and serious threat to public health. Previous studies have focused on the mechanisms of convergence of carbapenem resistance and hypervirulence (Lan et al., 2021), or focused only on hypervirulence strains (Lee et al., 2017), in the convergence of hypervirulence and carbapenem resistance in certain high-risk clones (Lai et al., 2019) or even in the molecular evolution mechanisms of carbapenem-resistant hvKp (Han et al., 2022), but none of them focused specifically on its virulence factors. Therefore, in this review, we summarize and discuss the virulence factors of carbapenem-resistant highly virulent or hypervirulent *K. pneumoniae* strains, as well as characterize the interplay of these virulence traits with the carbapenemase produced and sequence type of such strains.

## 2 Carbapenem resistance mechanisms of *Klebsiella pneumoniae*

Resistance to carbapenems in *K. pneumoniae* is associated with several mechanisms, including carbapenemase production, the co-occurrence of permeability defects together with the overproduction of β-lactamases with very weak carbapenemase activity, and the overexpression of efflux pumps (Pitout et al., 2015; Karampatakis et al., 2023). These mechanisms are briefly introduced in this review.

### 2.1 Carbapenemase production

Carbapenemase production is the most important mechanism among carbapenem-resistant *K. pneumoniae* strains. According to the Ambler classification, carbapenemases can be categorized into three classes: A, B, and D (Queenan and Bush, 2007). *Klebsiella pneumoniae* carbapenemases (KPCs) belong to the class A carbapenemases (those with serine in the active site) and are the most common carbapenemase found in carbapenem-resistant *K. pneumoniae* with over 90 KPC variants identified worldwide.^1^ KPC-1 (later shown to be identical to KPC-2) was first reported from a *K. pneumoniae* isolate in the United States in 1996 (Yigit et al., 2001) and remains the most common KPC variant today along with KPC-3 (Van Duin and Doi, 2017), both variants often carried by transposon Tn4401 (Nordmann et al., 2009). KPC is harbored in plasmids of different incompatibility (Inc) groups. The first KPC belonging to multilocus sequence types (ST) 258 was found in a pKpQIL-like plasmid, a type of plasmid that played a major role in the early dissemination of KPC-encoding elements (Yang et al., 2021a). KPC carbapenemase is known to hydrolyze β-lactams of all classes and several KPC variants are becoming resistant to new drug combinations such as ceftazidime-avibactam (Di Bella et al., 2021; Karampatakis et al., 2023). Guiana extended spectrum carbapenemase (GES) is another class A enzyme that was first identified in 2000 in a *K. pneumoniae* from French Guiana in a non-transferable pTK1 plasmid (Poirel et al., 2000). Although not all variants have activity against carbapenems, some have been found to hydrolyze imipenem efficiently: GES2, GES-4, GES-5, GES-6, GES-11, GES-14, and GES-18 (Nordmann and Poirel, 2014). Among these variants, GES-5 is the main carbapenem-hydrolyzing GES-type enzyme identified in *Enterobacteriaceae* in South America Nordmann and Poirel, 2014, particularly in Brazil (Picão et al., 2010; Ribeiro et al., 2014). In Europe, GES-5 have been identified in Portugal (Mendes et al., 2022b) and France (Bonnin et al., 2017) among *K. pneumoniae* strains. GES-1 and GES-5 seem to be often found in class 1 integrons, which is found on a considerable variety of plasmids among *K. pneumoniae* clones (Literacka et al., 2020).

Class B carbapenemase enzymes, also known as metallo-β-lactamases (MBLs), include primarily New Delhi metallo-β-lactamase (NDM), Verona integron-encoded metallo-β-lactamase (VIM), and imipenemase (IMP), and are characterized by the requirement of zinc at the active site (Behzadi et al., 2020). These enzymes hydrolyze a wide variety of β-lactams but cannot hydrolyze monobactams such as aztreonam (Behzadi et al., 2020). The genes encoding NDM, VIM, and IMP are usually plasmid-borne and can be transferred between bacterial strains (Queenan and Bush, 2007; Wu et al., 2019). New Delhi metallo-β-lactamase 1 (NDM-1) was first discovered in *K. pneumoniae* from a Swedish patient who traveled to New Delhi in 2008 (Yong et al., 2009). After the initial report of NDM-1, *bla*_NDM-1_ spread along the Indian subcontinent, including India, Pakistan, and Bangladesh, and has since spread globally (Wu et al., 2019). Several NDM variants have been identified worldwide (over 20), and at least 9 have been identified in *K. pneumoniae*. Furthermore, *Enterobactereaceae* are the major hosts of *bla*_NDM_, with *K. pneumoniae* accounting for just over half of all isolates identified (Wu et al., 2019). NDM was initially found within the Tn125 transposon, but have been more recently identified in Tn3000, with IncX3 being the most predominant replicon type found harboring this gene (Acman et al., 2022).

VIM-type enzymes were first identified in 1997 in Verona, Italy, from a *Pseudomonas aeruginosa* isolate (Lauretti et al., 1999) and subsequently spread worldwide, with a high prevalence in the southern part of Europe (Nordmann and Poirel, 2014). VIM-2 is one of the most reported MBLs worldwide, with an endemic spread in southern Europe (Italy, Spain, and Greece) and Southeast Asia (South Korea, Taiwan) (Nordmann and Poirel, 2014). VIM-1-producing *K. pneumoniae* has also been frequently reported in Greece (Nordmann and Poirel, 2014). VIM-type enzymes have been commonly identified in IncA/C, IncN or IncL/M plasmids harboring class 1 integron in *K. pneumoniae* strains (Sonnevend et al., 2017; Chavda et al., 2019; Cabanel et al., 2021). IMP-type β-lactamases were the first acquired MBL and were found in a *Serratia marcescens* isolate in Japan in 1991 (Ito et al., 1995). Several IMP variants have been identified, and IMP-type carbapenemase has spread worldwide, with endemic distributions reported in China, Japan, Taiwan, and Greece (Nordmann et al., 2012), although to a much lesser extent than KPC-, VIM-, NDM- or OXA-48-like enzymes (Nordmann and Poirel, 2014). IMP genes are often carried in class 1 integrons harbored on broad-host-range plasmids (Matsumura et al., 2017).

The most important class D carbapenemase is OXA-48 which was first reported in *K. pneumoniae* in Turkey in 2001 (Poirel et al., 2004). Since then, it has been reported from several countries, including Turkey, Morocco, Algeria, and India (Poirel et al., 2012). OXA-48 has several variants, including OXA-181, an enzyme reported mainly from the Indian subcontinent (Poirel et al., 2012) and one of the most identified variants of OXA-48-type enzymes worldwide (Pitout et al., 2020). Both enzymes have carbapenemase activity and their dissemination depends primarily on plasmids (Poirel et al., 2012). However, while OXA-48 is associated with different Tn1999 variants on IncL plasmids, OXA-181 is associated with IS*Ecp1*, Tn*2013* on ColE2, and IncX3 types of plasmids (Pitout et al., 2020).

### 2.2 Other mechanisms

**Figure 1:**
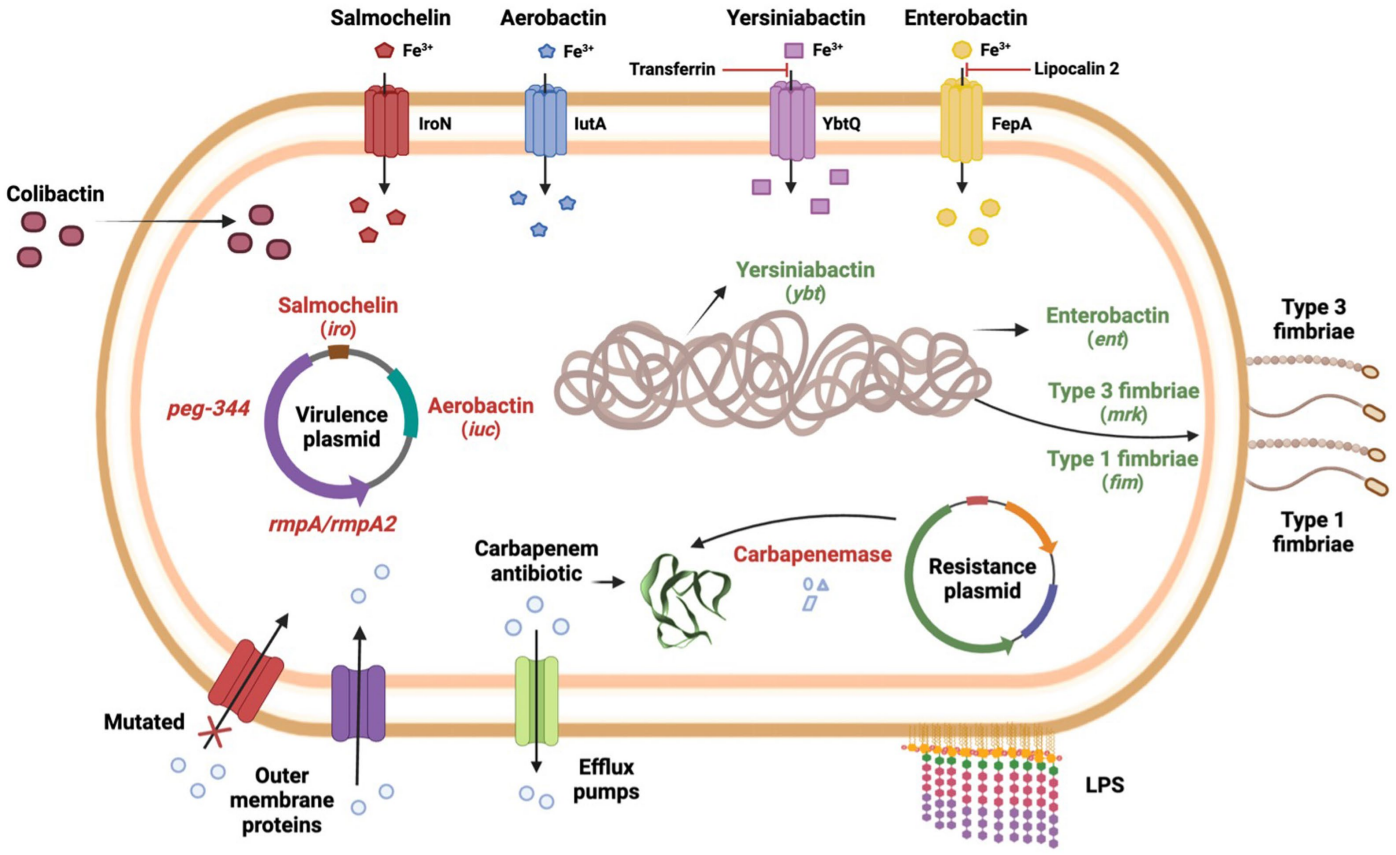
Carbapenem resistance mechanisms and virulence factors of carbapenem-resistant highly virulent or hypervirulent *K. pneumoniae*.

*Klebsiella pneumoniae* generally develops resistance through the production of carbapenem-hydrolyzing enzymes. However, the occurrence of permeability defects (e.g., porin alterations), the production of other β-lactamases with very weak carbapenemase activity, and the overexpression of efflux pumps represent alternative mechanisms in carbapenem-resistant *K. pneumoniae* (Pitout et al., 2015). However, it is worth noting that membrane permeability defects or efflux pump overexpression generally occur in pairs or in conjugation with carbapenemases/β-lactamases production. For example, although class C (AmpC-type β-lactamases) do not confer hydrolytic activity against carbapenems, they form a bond with the carbapenem molecule, preventing it from accessing its target (Suay-García and Pérez-Gracia, 2019). Furthermore, while carbapenemases specifically hydrolyze carbapenems and other β-lactams drugs, efflux pumps overexpression or membrane permeability defects are associated with multi-drug resistance in general (Suay-García and Pérez-Gracia, 2019). An overview of carbapenem resistance mechanisms is provided in Figure 1.

## 3 Virulence factors of *Klebsiella pneumoniae*

Once *K. pneumoniae* enters the host, it activates the host’s immune defenses, and therefore, to evade and cause infection, *K. pneumoniae* must overcome both mechanical and chemical barriers (Wang et al., 2020). To date, four major classes of virulence factors have been well characterized in *K. pneumoniae*: capsule, lipopolysaccharide (LPS), siderophores, and fimbriae (Paczosa and Mecsas, 2016; Wang et al., 2020). These virulence factors play different roles in different types of *K. pneumoniae* infections and different strains of *K. pneumoniae*, but ultimately, they are all strategies used by this bacterium to grow and protect itself from the host immune system (Paczosa and Mecsas, 2016).

### 3.1 Capsule

The capsule is a polysaccharide matrix that coats the cell and consists of strain-specific capsular polysaccharides, called K antigens. The capsule encoding genes are located on chromosomal operon *cps*, which contains several genes involved in capsule production, including *wzi*, *wza*, *wzb*, *wzc*, *gnd*, *wca*, *cpsB*, *cpsG*, and *galF*. K-antigen typing is often performed by sequencing the *wzi* locus which is strongly associated with specific K-antigens (Paczosa and Mecsas, 2016). To date, more than 160 capsular types have been identified (Lam et al., 2022), with K1 and K2 being the most prevalent capsular antigens in *K. pneumoniae* (Paczosa and Mecsas, 2016). In addition, K1 and K2 serotypes are more resistant to phagocytosis and host immune responses in general, thus making these K-antigens more virulent than strains of other serotypes (Paczosa and Mecsas, 2016).

In hvKp strains, a hypercapsule (also known as being hypermucoviscous), is usually produced, which significantly contributes to the pathogenicity of hvKp (Paczosa and Mecsas, 2016). The most common hvKp capsule types include K1 and K2 (accounting for approximately 70% of hvKp isolates) and to a lesser extent K5, K20, K54, and K57 (Russo and Marr, 2019).

### 3.2 Lipopolysaccharide

LPS, also known as endotoxin, is a component of the outer cell of Gram-negative bacteria. Although variable among bacterial species, LPS typically consists of an O-antigen, a core oligosaccharide, and lipid A, and can be an important virulence factor that protects against humoral defenses, while being a strong activator of an inflammatory response (Paczosa and Mecsas, 2016). Unlike K-antigen, only 9 different O-antigen serotypes and > 5 subtypes have been identified in *K. pneumoniae*, with serotypes O1 and O2 remaining the most common among clinical *K. pneumoniae* isolates (Follador et al., 2016; Wyres et al., 2020). Of note, the O1 antigen is usually associated with the K1 and K2 capsule types, making it the most common O-antigen type observed in hvKp strains (Russo and Marr, 2019).

Although the role of LPS could include protection against phagocytosis and enhanced virulence in general, studies have not found unique features in LPS produced by hvKp strains, suggesting that the enhanced virulence conferred by LPS is not specific to hvKp strains, but rather occurs in both hvKp and classical *K. pneumoniae* isolates (Russo and Marr, 2019).

### 3.3 Siderophores

Most organisms require iron as an essential element to survive and propagate during infection. *K. pneumoniae*, like many other organisms, must employ tactics to acquire iron from the host, as this element is limited and is not readily available in the host during infection (Miethke and Marahiel, 2007). *K. pneumoniae* secretes siderophores such as aerobactin, enterobactin, salmochelin, and yersiniabactin, which tightly bind to extracellular iron, and reenter the bacteria (Gonzalez-Ferrer et al., 2021). The content and expression of siderophores vary among different *K. pneumoniae* strains, but the ability to sequester iron is known to contribute to the pathogenic potential of this organism (Paczosa and Mecsas, 2016).

Enterobactin is almost ubiquitous in *K. pneumoniae* and is the primary iron uptake system used by this pathogen, despite being inactivated by the host protein lipocalin-2 (Flo et al., 2004; Paczosa and Mecsas, 2016; Russo and Marr, 2019). Enterobactin biosynthesis is carried chromosomally by the *entABCDEF* gene cluster, and the proteins that mediate its transport are carried by the *fepABCDG* gene cluster, with *fepA* specifically encoding the Fe^3+^ uptake receptor (Paczosa and Mecsas, 2016). Yersiniabactin, first reported in Yersinia, is a siderophore encoded by the *irp* genes, and the transporters required for its secretion are encoded by the *ybt* and *fyu* genes, while the *ybtQ* is thought to encode the uptake receptor for yersiniabactin. This siderophore is not inhibited by lipocalin-2 but is unable to acquire iron in the presence of transferrin (Paczosa and Mecsas, 2016). Salmochelin is a glycosylated derivative of enterobactin, and its synthesis, excretion, and uptake require five genes, *iroBCDEN*, with *iroN* encoding the salmochelin receptor (Müller et al., 2009). Furthermore, salmochelin is not inhibited by lipocalin-2, which confers an advantage over enterobactin in lipocalin-2-sufficient hosts (Fischbach et al., 2006). Aerobactin is a citrate-hydroxamate siderophore and its synthesis is encoded by the *iucABCD* gene together with its transporter *iutA*. Importantly, in terms of iron binding efficiency, aerobactin appears to be less advantageous when compared to other siderophores (lower affinity with different iron sources) (Miethke and Marahiel, 2007).

### 3.4 Fimbriae

Type 1 and type 3 fimbriae represent another class of virulence factors and are primarily responsible for *K. pneumoniae* adhesion to biotic and abiotic surfaces. Type 1 fimbriae are responsible for adhesion to mannose-containing structures on both host cells and the extracellular matrix and are found among several members of the *Enterobacteriaceae* (Struve et al., 2008).

The *fimA* gene encodes the subunit *FimA* (body structure) and *fimH* encodes the subunit *FimH* (tip structure), which is responsible for the adhesive properties (Paczosa and Mecsas, 2016). Additionally, *fimK* is a unique gene in *K. pneumoniae* that belongs to the type 1 fimbriae gene cluster and is thought to play a role in regulating fimbrial expression (Struve et al., 2008). Moreover, type 1 fimbriae are expressed in the urinary tract but not in the gastrointestinal tract or lungs (Struve et al., 2008). Type 3 fimbriae are encoded by the *mrkABCD* gene cluster, consisting of a MrkA subunit as the main structure and a MrkD subunit as the tip (Murphy and Clegg, 2012; Ahmadi et al., 2021). In contrast to type 1 fimbriae, type 3 fimbriae do not bind mannose, but have been shown to bind extracellular matrix proteins such as collagens (Murphy and Clegg, 2012). While the genes encoding for type 3 fimbriae production in *K. pneumoniae* may be either plasmid-borne or carried in the chromosome, the *mrk* genes are usually detected on conjugative plasmids and/or transposons in *Escherichia coli* strains (Murphy and Clegg, 2012; Khonsari et al., 2021) and are therefore not frequently observed in this bacterium (Khonsari et al., 2021).

Biofilm formation has been proposed to contribute to bacterial resistance and virulence since the bacteria are less susceptible to being killed by innate host defense mechanisms and are less susceptible to the action of many antibiotics. Therefore, biofilm production is arguably among the most clinically relevant roles of fimbriae. For example, type III fimbriae are required for *K. pneumoniae* biofilm production and attachment to medical devices (Schroll et al., 2010; Stahlhut et al., 2012). In addition, while one study found that type I fimbriae mediate binding, invasion, and biofilm formation in the bladder during *K. pneumoniae* urinary tract infections (Rosen et al., 2008), another study suggests that type 1 fimbriae do not promote biofilm production in *K. pneumoniae* (Schroll et al., 2010). Thus, type 3 fimbriae are crucial for biofilm production, whereas the role of type 1 fimbriae in biofilm production by *K. pneumoniae* is still controversial. On the other hand, the expression of fimbriae by *K. pneumoniae* also increases their binding to phagocytes, triggering phagocytosis, which is likely to increase the clearance of *K. pneumoniae*, thus compromising the virulence of this pathogen (Paczosa and Mecsas, 2016).

### 3.5 Colibactin

First described in *E. coli* harboring polyketide synthase (*pks*) genes, colibactin is a peptide-polyketide hybrid genotoxin capable of causing cross-links of DNA and disrupting host immune responses (Faïs et al., 2018). The *pks* island hosts 19 genes for colibactin synthesis (*clbA*-*clbR*) and shares a 100% sequence identity with *K. pneumoniae* (Strakova et al., 2021). In *K. pneumoniae*, the *pks* genomic island is encoded on the chromosomal integrative and conjugative element of *K. pneumoniae* 10 (ICE*Kp*10) (Lam et al., 2018). Also, it is speculated that the *pks* locus promotes gut colonization and mucosal invasion leading to enhanced transmissibility and virulence in *K. pneumoniae* (Strakova et al., 2021). The pks cluster was first detected in *K. pneumoniae* in 2009, and among 141 strains studies, only 3.5% were *pks* positive strains (Putze et al., 2009). A further study conducted in China showed that among 190 *K. pneumoniae* bloodstream strains, 27% were *pks* positive isolates (Lan et al., 2019). More recently, a study conducted in Portugal detected the colibactin gene *clb* encoded on ICE*Kp*10 in 24% of the carbapenemase-producing *K. pneumoniae* strains studied (Mendes et al., 2022b).

## 4 Hypervirulence: epidemiology and associated factors

Since the 1980s and 1990s, strains of *K. pneumoniae* that can cause serious infections in otherwise healthy individuals have received worldwide attention. Reports from Taiwan described a unique clinical syndrome of community-acquired *K. pneumoniae* infection, causing pyogenic liver abscesses in otherwise healthy individuals with metastatic spread to distant sites and lacking a history of hepatobiliary disease (Liu et al., 1986; Pomakova et al., 2012; Shon et al., 2013). To distinguish this pathotype from classical *K. pneumoniae* infections, the term ‘hypervirulent’ *K. pneumoniae* was therefore introduced. In 2004, a study demonstrated that *K. pneumoniae* strains causing hepatic abscesses in Taiwanese patients were more likely to have a hypermucoviscous phenotype than non-invasive strains (Fang et al., 2004), and as a result, hvKp strains were often referred to as hypermucoviscous in the literature. Phenotypically, hypermucoviscosity is defined by the “string test,” which consists of the formation of viscous strings with >5 mm in length when a loop is used to stretch the colony on an agar plate (Fang et al., 2004). In addition, several factors can enhance capsule production (regulate hypermucoviscosity), including mucoviscosity-associated gene A (*magA*), regulator of mucoid phenotype A (*rmpA*), and *rmpA2* and the Rcs two-component system (*rcsAB*). Importantly, hypermucoviscous and hypervirulent have often been used synonymously in the literature, but it is now known that not all hypervirulent strains exhibit the hypermucoviscous phenotype, and not all hypermucoviscous strains lead to an invasive syndrome (Choby et al., 2020).

Most of the infections caused by hvKp have been identified mainly in the Pacific Rim countries and usually occur in specific sequence types, mainly ST23 but also ST65, and ST86, among many others (Russo and Marr, 2019). Notably, a recent study showed that CG23 evolved around 1878, years before the first description of *K. pneumoniae* by Carl Friedlander, further supporting the idea that Friedlander’s bacillus may have been the first description of an hvKp strain (Lam et al., 2018). Furthermore, this study also showed that most human clinical strains of CG23 belong to CG23-I, a clonally expanded sublineage that emerged in 1928 and has undergone particularly accelerated population growth and global distribution. This sublineage is also strongly associated with liver abscess infections, demonstrating that the circulation of CG23 began decades before the first reports in the mid-1980s.

Although usually associated with pyogenic liver abscesses, hvKp can also cause extrahepatic diseases such as pneumonia, urinary tract infections, kidney and lung abscesses, and other types of infections (Choby et al., 2020). In addition, hvKp infections are seen in all age groups and otherwise healthy individuals, although some patients have comorbidities (e.g., diabetes; Shon et al., 2013). Furthermore, while hvKp is more commonly associated with community-acquired diseases, there have been increasing reports of hvKp causing healthcare-associated diseases, including an outbreak of a carbapenem-resistant ST11 hvKp in a Chinese hospital that caused a fatal ventilator-associated pneumonia in five infected patients (Gu et al., 2018). As a result of all these adaptive and evolutionary features, it is not surprising that infections caused by hvKp have emerged worldwide.

Currently, approximately 40 years after the first reports, the definition of hypervirulence is still not agreed upon in the literature, as there seems to be no single categorization that can encompass all hvKp strains. Clinically, hepatic abscess in the absence of biliary tract disease is the most common underlying disease caused by hvKp, but these strains can cause a variety of other diseases as well. Phenotypically, the “string test” is also an important indicator of hypervirulence, as most hvKp also exhibit the hypermucoviscosity phenotype, but this test performed only moderately well as a predictor of classical *K. pneumoniae* vs. hvKp (Russo et al., 2018). Genetically, several traits are suggestive of hypervirulence in *K. pneumoniae*. K1 and K2 capsular antigen types and O1 antigen type are commonly observed in hvKp strains (Russo and Marr, 2019), but alone cannot predict the hypervirulent pathotype. Similarly, salmochelin and aerobactin are highly prevalent among hvKp, with one study even suggesting that aerobactin is a critical virulence factor that enhances virulence *ex vivo* and *in vivo* (Russo et al., 2015), but alone cannot predict hvKp strains. Thus, rather than a specific gene or trait, a combination of traits is the best way to predict hypervirulence in *K. pneumoniae*. Importantly, one study showed that biomarkers encoded on the virulence plasmid (i.e., pK2044 and pLVPK) are considered to be the most accurate in differentiating hvKp from classical *K. pneumoniae* strains. These biomarkers included the *peg-344*, *iroB*, *iucA*, and plasmid-encoded *rmpA*, and *rmpA2* genes (Russo et al., 2018). A combination of these biomarkers is currently considered the best option for predicting hvKp strains and should be encouraged to be used as a tool for rapid assessment in healthcare settings. Additionally, pathogenicity and virulence assays, preferably in murine infection models (rather than *G. mellonella* models), could help to better differentiate hypervirulent from classical *K. pneumoniae* (Russo and Macdonald, 2020). An overview of the virulence factors mentioned above can be visualized in Figure 1.

## 5 Selection criteria for the virulence factors included in this review

Relevant studies containing detailed descriptions of key virulence factors, carbapenem resistance genes, and clonal information were included. As a follow-up to what was discussed above, we considered the major classes of virulence factors that are better characterized in *K. pneumoniae* (Paczosa and Mecsas, 2016) as well as other important virulence factors that help to better differentiate hypervirulent *K. pneumoniae* strains from classical strains (i.e., *rmpA*, *rmpA2,* and *peg-344*) (Russo et al., 2018).

## 6 Global distribution of virulence factors in carbapenem-resistant highly virulent or hypervirulent *Klebsiella pneumoniae*

**Figure 2:**
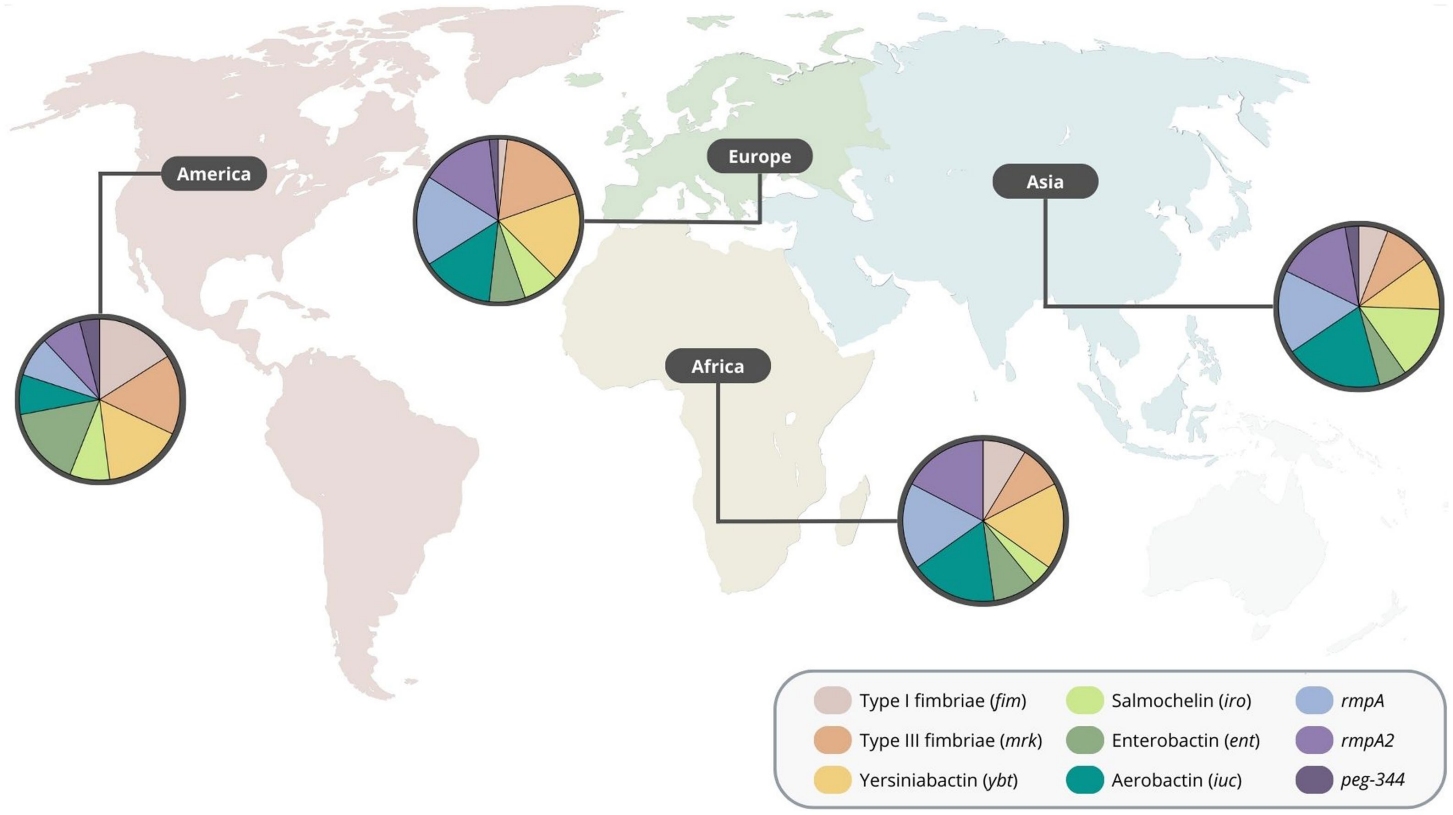
Distribution of virulence genes of carbapenem-resistant highly virulent or hypervirulent *Klebsiella pneumoniae* by region.

The Asian continent was by far the region with more reports of carbapenem-resistant highly virulent or hypervirulent *K. pneumoniae* (Table 1; Supplementary Table S1). In this continent, aerobactin was the most common virulence gene detected, followed by *rmpA*, *rmpA2* and salmochelin. Various reports from Europe were also identified, where *rmpA*, yersiniabactin, type 3 fimbriae, aerobactin, and *rmpA*2 were the most common. Additionally, type 1 and type 3 fimbriae, yersiniabactin and enterobactin were the most common virulence genes detected in the American continent. Moreover, reports from the African continent showed that the aerobactin, yersiniabactin, *rmpA* and *rmpA2* were among the most detected virulence genes (Figure 2), indicating an overall different distribution of virulence traits between continents. Regarding the number of virulence genes per region, the African, American. European and Asian continents had an average of 4.6, 4.2, 4.7, and 4.5 virulence genes, respectively (Supplementary material).

**Table 1:** Summary of worldwide reports of carbapenem-resistant highly virulent or hypervirulent *Klebsiella pneumoniae*, grouped by region.

Continent	Country	Year of Isolation	ST	Virulence genes	Serotype	Carbapenem resistance genes	Virulence/Resistance MGE	String test	Reference
Africa	Egypt	2017-2018	ST11	*fimABCDFGHIK*, *mrkABCDFJIH*, *fyuA*, *ybtAEPQSTUX*, *irp1*, *irp2*, *iroE*, *entABCDE*, *fepABCDG*, *fes*, *ybdA*, *iucABCD*, *iutA*, *rmpA*, *rmpA2*, *rcsAB*	K47	NDM-1 + KPC-2	pEBSI036-1-NDM-VIR, pEBSI036-2-KPC	Unknown	Ahmed et al. (2021)
Africa	Egypt	2018	ST11	*ybt*, *iuc*, *rmpA*, *rmpA2*	K1	NDM-1 + KPC-2	Unknown	Unknown	Sherif et al. (2021)
Africa	Egypt	2016	ST1399	*fimABCDFGH*, *mrkABCDF*, *entAB*, *fepC*, *iutA*, *rcsAB*	K43	NDM-1	p2	Unknown	Abdelwahab et al. (2021)
America	Brazil	2014-2016	ST11	*fimABCDFGH*, *mrkABCDFJ*, *ybt*, *ent*	K64	KPC-2	Unknown	Unknown	Andrey et al. (2020)
America	United States of America	2016	ST23	*iroBCDN*, *iucABCD*, *iutA*, *rmpA*, *rmpA2*, *peg-344*	K1	KPC-2	pDHQP1701672_hv; pDHQP1701672_amr	Negative	Karlsson et al. (2019)
America	Canada	2018	ST86	*fyuA*, *ybtAEPQSTUX*, *irp1*, *irp2*, *iroBCDN*, *iucABCD*, *iutA*, *rmpA*, *rmpA2*	K2	KPC-2	phvKP060; pKPCKP060	Positive	Mataseje et al. (2019)
Asia	China	2022*	ST11	*mrkACDF*, *ybtAEPQSTUX*, *iroEN*, *iutA*	K47	KPC-2	~ JX-CR-hvKP-2-P2	Negative	Cai et al. (2022)
Asia	China	2020	ST11	*iucABCD*, *iutA*, *rmpA*, *rmpA2*	K64	KPC-2	p3_L39; pKp58-1	Unknown	Chen et al. (2021)
Asia	China	2015-2018	ST11	*ybt*, *iuc*, *rmpA*	K47	KPC-2 + NDM	pLVKP-like plasmid	Unknown	Cienfuegos-Gallet et al. (2022)
Asia	China	2016	ST11	*mrkABCDFHIJ*, *fyuA*, *ybtAEPQSTUX*, *irp1*, *irp2*	K64	KPC-2	pKPC2_020002	Negative	Feng et al. (2019)
Asia	China	2016	ST11	*fimABCDFGHLK*, *mrkABCDFHIJ*, *irp1*, *irp2*, *iroE*, *ent*, *iucABCD*, *iutA*, *rmpA2*	K47	KPC-2	pLVPK-like plasmid; pVir-CR-HvKP4-like	Positive	Gu et al. (2018)
Asia	China	2020	ST11	*fimABCDEFGHIK*, *mrkABCDFHIJ*, *fyuA*, *ybtAEPQSTUX*, *irp1*, *irp2*, *iroEN*, *entABCDEFS*, *fepABCDG*, *fes*, *iucABCD*, *iutA*, *rmpA2*, *rcsAB*	K64	NDM-5 + KPC-2	pB; pC	Positive	Hu et al. (2021)
Asia	Taiwan	2012-2014	ST11	*fimABCDEFGHIK*, *mrkABCDFHIJ*, *irp1*, *irp2*, *iroBCDEN*, *entB*, *iucABCD*, *iutA*, *rmpA*, *rmpA2*	K47	KPC-2	TVGHCRE225 pVir; pKPC-LK30-like	Positive	Huang et al. (2018)
Asia	Pakistan	2021*	ST11	*mrk*, *fyuA*, *ybtAEPQSTUX*, *irp1*, *irp2*, *fep*, *iucABCD*, *iutA*, *rmpA2*	K24	NDM-1	pLVKP-like plasmid	Negative	Imtiaz et al. (2021)
Asia	China	2016-2018	ST11	*fyuA*, *ybtAEPQSTUX*, *irp1*, *irp2*, *iroBCDN*, *iucABCD*, *iutA*, *rmpA*, *rmpA2*, *peg-344*	K64	KPC-2	pLVKP-like plasmid	Unknown	Kong et al. (2021)
Asia	China	2019-2020	ST11	*iuc*, *rmpA*, *rmpA2*, *peg-344*	K64	KPC-2	Unknown	Negative	Li et al. (2022)
Asia	China	2018	ST11	*fim*, *mrk*, *irp-1*, *iroN*, *ent*, *iucA*, *rmpA*, *rmpA2*, *peg-344*	K64	KPC-2	Unknown	Negative	Ouyang et al. (2022)
Asia	China	2019	ST11	*fimH*, *mrkD*, *iucB*, *rmpA*	K57	KPC-2	Unknown	Negative	Shao et al. (2021)
Asia	China	2016-2017	ST11	*iroBCD*, *iucABCD*, *rmpA2*	K64	KPC-2	pLVPK-like plasmid	Negative	Sun et al. (2019)
Asia	China	2020	ST11	*iroN*, *iucA*, *rmpA*, *rmpA2*, *peg-344*	K64	KPC-2	Unknown	Positive	Wei et al. (2022)
Asia	China	2011-2017	ST11	*iroN*, *iucA*, *iutA*, *rmpA*, *rmpA2*	K64	KPC-2	pLVPK-like plasmid	Positive	Xu et al. (2019)
Asia	China	2016	ST11	*mrkABCDFHIJ*, *ybt*, *iucABCD*, *iutA*, *rmpA2*	K47	KPC-2	Tn7074; p16HN-263_KPC	Negative	Yang et al. (2020)
Asia	China	2015	ST11	*ybt*, *iucABCD*, *iutA*, *rmpA2*	K47	KPC-2	pSH12_Vir; pSH12_KPC; ICE*Kp*3	Negative	Yang et al. (2021b)
Asia	China	2019	ST11	*fyuA*, *ybtAEPQSTUX*, *irp1*, *irp2*, *iroBCDN*, *entABCDEFS*, *fepABCDG*, *fes*, *iucABCD*, *iutA*, *rmpA*, *rmpA2*	K64	KPC-2	pKP20194a-p2; pKP20194a-p1	Positive	Zhang X. et al. (2020)
Asia	China	2017	ST11	*iroN*, *iucA*, *rmpA*, *rmpA2*	K64	KPC-2	Unknown	Negative	Zhang et al. (2020b)
Asia	China	2015	ST14	*fimH*, *mrkD*, *iuc*, *rmpA*	K2	NDM-5	p24835-NDM5	Positive	Mei et al. (2017)
Asia	Singapore	2013-2015	ST23	*ybt*, *iro*, *iuc*, *rmpA*, *rmpA2*	K1	KPC-2	pKPC2	Positive	Chen et al. (2020)
Asia	China	2013	ST23	*iroBCDN*, *iucABCD*, *iutA*, *rmpA*, *rmpA2*	K1	KPC-2	pKP70-2	Positive	Dong et al. (2018)
Asia	China	2018	ST23	*mrkABCDFJIH*, *fyuA*, *ybtSXQPA*, *irp1*, *irp2*, *iroBCDN*, *iucABCD*, *iutA, rmpA*, *rmpA2*	K1	VIM-1	pR210-2-VIM; pR210-2-vir	Positive	Dong et al. (2019)
Asia	Russia	2019	ST23	*fimH*, *mrk*, *fyuA*, *ybt*, *irp*, *ent*, *iuc*, *iut*, *rmpA*, *peg-344*	K57	OXA-48 + NDM-1	Unknown	Positive	Fursova et al. (2022)
Asia	China	2019*	ST23	*iroBCDN*, *iucABCD*, *iutA*, *rmpA*, *rmpA2*	K1	NDM-1	pNDM_3214; pVIR_3214	Positive	Liu and Su (2019)
Asia	Singapore	2011-2015	ST23	*ybt*, *iro*, *iuc*, *rmpA*, *rmpA2*	K1	KPC-2	Unknown	Unknown	Octavia et al. (2019)
Asia	Russia	2014	ST23	*mrkABCDFHIJ*, *fyuA*, *ybtAEPQSUX*, *irp1*, *iroBCDN*, *iucABCD*, *rmpA*, *rmpA2*	K1	OXA-48	pOXA-48-like; pSGH10-like	Unknown	Shaidullina et al. (2020)
Asia	Iran	2012	ST23	*iuc*, *rmpA*	K1	VIM-2	Unknown	Positive	Tabrizi et al. (2018)
Asia	China	2011-2017	ST23	*iroN*, *iucA*, *iutA*, *rmpA*, *rmpA2*	K1	KPC-2	pLVPK-like plasmid	Positive	Xu et al. (2019)
Asia	China	2019-2020	ST23	*fimH*, *mrkD*, *entB*, *iucA*, *iutA*, *rmpA*, *rmpA2*	K1	KPC-2	Unknown	Positive	Zhou et al. (2021)
Asia	China	2018	ST29	*mrkABCDFHIJ*, *fyuA*, *ybtAEPQSTUX*, *irp1*, *irp2*, *iroBCDEN*, *entABCDEFS*, *fepABCDG*, *iucABCD*, *iutA*, *rmpA*	K54	NDM-5	pVir-SCNJ1; pNDM5-SCNJ1	Positive	Yuan et al. (2019)
Asia	China	2015	ST36	*mrkABCDFJIH*, *fyuA*, *ybtSXQPA*, *irp1*, *irp2*, *iroBCDN*, *iucABCD*, *iutA*, *rmpA*, *rmpA2*	K62	KPC-2	pVir-KP13F2; pKPC-KP12F2	Negative	Feng et al. (2018)
Asia	Japan	2006-2017	ST65	*iro*, *iuc*, *rmpA*, *rmpA2*	K2	IMP-6	pLVKP-like plasmid; pKPI-6-like plasmid	Positive	Le et al. (2022)
Asia	China	2014-2019	ST65	*iroN*, *iucABCD*, *iutA*, *rmpA*, *rmpA2*	K2	IMP-4	pVir-C1672; pRes-C1672	Negative	Zhang et al. (2021)
Asia	China	2017	ST86	*iroN*, *iucABCD*, *iutA*, *rmpA2*	K2	NDM-1 + KPC-2	p30457-1; p30457-3; p30457-4	Positive	Liu et al. (2019)
Asia	China	2017	ST86	*fimABCDFGHIK*, *mrkABCDFJIH*, *iroBCDEN*, *entABCDEFS*, *fepABCDG*, *iutA*, *rmpA*	K2	KPC-2	pK55602_1; pK55602_2	Negative	Liu et al. (2020)
Asia	China	2017	ST86	*mrkABCDFHIJ*, *iroBCDN*, *iucABCD*, *iutA*, *rmpA*, *rmpA2*	K2	KPC-2	p17ZR-91-Vir; p17ZR-91-KPC	Positive	Xie et al. (2021)
Asia	Iran	2017-2018	ST147	*ybtS*, *iucA*, *rmpA*	K2	OXA-48 + NDM-1	Unknown	Positive	Solgi et al. (2020)
Asia	China	2015-2017	ST375	*fimABCDEFH*, *mrkD*, *iroBD*, *entAB*, *iucBC*, *iutA*, *rmpA*	K2	KPC-2	ptig00000041; ptig00000014	Positive	Su et al. (2020)
Asia	China	2021	ST464	*fimABCDEFGHIK*, *mrkABCDFHIJ*, *iroE*, *entABCDEFS*	K53	NDM-1 + KPC-2	pSW25KPC2; pSW25NDM1	Negative	Hao et al. (2022)
Asia	China	2019	ST592	*iroBCDN*, *iucABCD*, *iutA*, *rmpA*, *rmpA2*	K57	KPC-2	pVir_090515; pKPC-2_090515	Negative	Zhao et al. (2022)
Asia	China	2019-2020	ST859	*fimABCDFGH*, *mrkABCDF*, *iroE*, *entABCDEFS*, *fepABCDG*, *fes*, *ybdAi*, *iucABCD*, *iutA*, *rmpA*, *rmpA2*	K19	KPC-2	pVir-RJ9299; pKPC-2-RJ9299	Positive	Zhu et al. (2022)
Asia	China	2014	ST1265	*mrkABCDFHIJ*, *fyuA*, *ybtAEPQSTUX*, *irp1*, *irp2*, *iroBCDEN*, *iucABCD*, *iutA*, *rmpA*, *rmpA2*	K1	KPC-2	p11420-vir; p11420-KPC	Positive	Li et al. (2020)
Asia	China	2013-2017	ST1660	*ybt*, *iro*, *iuc*, *rmpA*, *rmpA2*, *peg-344*	K1	KPC-2	Unknown	Positive	Shen et al. (2020)
Europe	Portugal	2019	ST13	*fimABCDFGHIK*, *mrkABCDFHIJ*, *ybtAEPQSTUX*, *irp1*, *irp2*, *fyuA*, *iroEN*, *entABCDEFS, fepABCDG*, *fes*, *iutA*	K3	KPC-70	Unknown	Positive	Mendes et al. (2022a)
Europe	France	2017*	ST23	*mrkD*, *ybtS*, *entB*, *iutA*, *rmpA*	K1	OXA-48 + NDM-1	Unknown	Positive	Compain et al. (2017)
Europe	Spain	2021*	ST23	*mrkABCDFHIJ*, *fyuA*, *ybtAEPQSTUX, irp1*, *irp2*, *iroBCDN*, *iucABCD*, *iutA*, *rmpA*, *rmpA2*	K1	OXA-48	pMS3802-CTXM-vir; pMS3802OXARMA	Negative	Hernández et al. (2021)
Europe	United Kingdom	2015	ST23	*ybtAEUPQRSTX*, *iroBCDN*, *iucABCD*, *iutA*, *rmpA2*	K1	NDM-1	Unknown	Unknown	Roulston et al. (2018)
Europe	France	2017	ST86	*ybt*, *iroBCDN*, *iucABCD*, *iutA*, *rmpA*, *rmpA2*	K2	OXA-48	pVIR-Kpn154; ICE*Kp*3	Positive	Beyrouthy et al. (2020)
Europe	Italy	2019-2020	ST147	*mrkABCDF*, *ybt*, *iuc*, *rmpA*, *rmpA2*, *peg-344*	K64	NDM-1	pSI0739-ARMA-Vir; pSI0739-NDM	Unknown	Martin et al. (2021)
Europe	Italy	2019	ST383	*mrkABCDF*, *iucABCD*, *iutA*, *rmpA*, *rmpA2*	K51	OXA-48 + NDM-1 + NDM-5	IncL_OXA48; IncA/C2_NDM-1; IncFIB(pNDM-Mar)/IncHI1B(pNDM-MAR)_NDM5	Positive	Lorenzin et al. (2022)

Strains within the same region are ordered by sequence type.

It is reasonable to highlight, however, that carbapenem-resistant highly virulent or hypervirulent *K. pneumoniae* strains are currently underdetected worldwide (Bonnin et al., 2020; Mendes et al., 2022b), particularly in African, Oceania and American countries (Russo and Marr, 2019; Choby et al., 2020; Arcari and Carattoli, 2023). Additionally, in Europe (more specifically in the EU/EEA region), the detection of virulence genes is not part of diagnostic microbiology routines, which affects the overall detection of these strains in this region (European Centre for Disease Prevention and Control, 2021).

## 7 Interplay between antimicrobial resistance and virulence factors in carbapenem-resistant highly virulent or hypervirulent *Klebsiella pneumoniae*

Over the years, *K. pneumoniae* has demonstrated the ability to easily acquire genetic elements and mutations that confer antimicrobial resistance and/or virulence traits. These acquisitions have led to the emergence and convergence of *K. pneumoniae* harboring both antimicrobial resistance and virulence genes. In some cases, and depending on the resistance genes acquired, these strains may be resistant to only a few antibiotics or multidrug-resistant–resistant to at least one antibiotic from three different antimicrobial classes (penicillins, cephalosporins, carbapenems, monobactams, tetracyclines, macrolides, fluoroquinolones, aminoglycosides, sulfonamides, among others), which is very common worldwide (Wang et al., 2020). In terms of virulence genes and depending on which virulence genes are acquired by the bacteria, *K. pneumoniae* strains can be classified as virulent or hypervirulent, although the definition of hypervirulence is still not clear and consensual worldwide. Overall and in the worst-case scenario, the convergence of both antimicrobial resistance and virulence genes in the same bacteria might culminate in MDR-hvKp (Tang et al., 2020). The acquisition of antimicrobial resistance and virulence traits is mainly driven by horizontal gene transfer mediated by mobile genetic elements, including transposons, integrons, plasmids, insertion sequences, and ICEs, and can occur when MDR-*K. pneumoniae* acquire hypervirulent plasmids (e.g., pK2044, pLVPK), hvKp strains obtain MDR plasmids (generally conjugative plasmids), and the acquisition of hybrid plasmids harboring virulence and resistance determinants (Tang et al., 2020).

In recent years, there has been an exponential increase in the number of reports of highly virulent or hypervirulent carbapenem-resistant *K. pneumoniae* (Table 1; Supplementary Table S1). The majority of *K. pneumoniae* strains observed in this review harbored the *bla*_KPC-2_ gene since it is the most common carbapenemase in China among carbapenem-resistant hvKp strains (Zhang Y. et al., 2020) and most studies included in this review were from China. In 2016, an outbreak of ST11-K47 KPC-2-producing hvKp strains was reported in a Chinese hospital causing fatal ventilator-associated pneumonia in five infected patients (Gu et al., 2018). These isolates were carrying both *bla*_KPC-2_-bearing plasmid and a pLVPK-like virulence plasmid harboring *iucABCD*, *iutA*, *iroBCDN*, *rmpA,* and *rmpA2* genes. The authors conducted several assays, including a human neutrophil assay and virulence potential assays in *Galleria mellonella* models, and all ST11 carbapenem-resistant *K. pneumoniae* outbreak strains showed high virulence.

More recently, a retrospective multicenter study conducted in China revealed the presence of 55 carbapenem-resistant hvKp strains, 44 of which were ST11-K64 KPC-2-producing strains (Zhang Y. et al., 2020). The authors conducted a fitness analysis assay using three ST11-K64 KPC-2-producing hvKp and one classical ST11 KPC-2-producing *K. pneumoniae* strain and found no significant differences between them, suggesting no compromising fitness cost and potential dissemination risk for carbapenem-resistant hvKp strains. KPC-2-producing hypervirulent *K. pneumoniae* have also been reported in other countries, including an ST86-K2 *K. pneumoniae* in Canada carrying a hypervirulent plasmid (phvKP060) and a KPC-2 plasmid (pKPCKP060) (Mataseje et al., 2019), an ST23-K1 *K. pneumoniae* in the United States of America carrying pDHQP1701672_hv and pDHQP1701672_amr plasmids (Karlsson et al., 2019), and an ST11-K47 *K. pneumoniae* strain in Taiwan carrying an TVGHCRE225 pVir plasmid and an IncR/IncFII/IncN plasmid harboring KPC-2 (Huang et al., 2018).

Another study conducted in China reported two ST65-K2 IMP-4-producing *K. pneumoniae* strains carrying the *iroN*, *iucABCD*, *iutA*, *rmpA,* and *rmpA2* genes in a pLVPK-like virulence plasmid belonging to the IncHI1B/IncFIB replicon (Zhang et al., 2021). The *bla*_IMP-4_ was located in IncU/N plasmids. Since IMP-producing MDR-hvKp are not common in China, the authors evaluated the stability of the resistance plasmid and the fitness of the strains after the acquisition of resistance plasmids and concluded that the stability of the resistance plasmid was high (87 and 93.7%) and the plasmid-cured mutants presented similar growth rates when compared to the parental strains. Also, the strains demonstrated high virulence in *G. mellonella*, suggesting that these two IMP-4-producing hypervirulent *K. pneumoniae* strains have the potential to evolve and spread without compromising fitness. Furthermore, a study conducted in Japan reported 104 IMP-1/IMP-6 producing *K. pneumoniae* strains, including four ST23-K1 isolates, 10 ST65-K2 isolates, and 7 ST86-K2 isolates harboring multiple virulence genes, including *rmpA*, *rmpA2*, *iutA,* and *iroN*, suggesting the emergence of IMP-1/6 producing hvKp strains in Japan (Yonekawa et al., 2020).

In 2018 a retrospective study conducted in China led to the identification of an ST23-K1 VIM-1-producing hvKp strain carrying various virulence genes, including *mrkABCDFJIH*, *ybtSXQPA*, *iucABCD*, *iutA*, *iroBCDN*, *rmpA* and *rmpA2* encoded in a IncHI1B/IncFIB_Қ_ plasmid (Dong et al., 2019). The virulence level of these strains was tested in wax moth (*G. mellonella*) larvae and a mouse infection model and showed high mortality rates in both assays. Furthermore, the authors performed conjugation experiments to test the transferability of the IncA group plasmid pR210-2-VIM using a rifampin-resistant *E. coli* EC600 (Rifr) strain as the recipient, and the transconjugant also showed resistance to most antibiotics tested, further supporting the convergent evolution of MDR-hvKp with high dissemination capacities and with minimal fitness costs. Of concern, a fatal outbreak of hvKp harboring *bla*_VIM-2_ located in a IncN plasmid was reported in 2012 among mechanically ventilated patients with drug intoxication in Iran (Tabrizi et al., 2018). These isolates belonged to the high-risk clone ST23-K1 and carried virulence genes such as aerobactin and *rmpA* in a. In addition, the *bla*_VIM-2-_carrying plasmid in VIM-producing *K. pneumoniae* strains was successfully transferred to the recipient strain *E. coli* K12, further highlighting the easy horizontal dissemination of this carbapenemase gene in hvKp strains that can cause fatal outbreaks.

In 2017, an ST86-K2 OXA-48-producing *K. pneumoniae* strain was recovered from the urine of a patient with a community-acquired urinary tract infection in France (Beyrouthy et al., 2020). The isolate also carried the virulence genes *rmpA*, *rmpA2*, *iucABCD*, *iutA,* and *iroBCDN* within a IncHI1B/IncFIB virulence plasmid replicon and showed high mouse lethality (like NTUH-2044). More recently, other studies have reported ST23-K1 OXA-48 producing hvKp in Russia (Shaidullina et al., 2020; Fursova et al., 2022), and Spain (Hernández et al., 2021). A study from the United Kingdom also revealed the presence of an ST23-K1 OXA-48 producing hvKp strain carrying the genes *iroBCDN*, *iucABCD*, *iutA*, *mrkABCDF*, *ybtAEPQSTUX*, *rmpA* and *rmpA2* (Turton et al., 2018).

ST23-K1 NDM-1-producing *K. pneumoniae* has been reported in the United Kingdom (Roulston et al., 2018), Saudi Arabia (Compain et al., 2017), and China (Liu and Su, 2019). NDM-5-producing *K. pneumoniae* was also identified in an ST14-K2 strain from China (Mei et al., 2017). The NDM-5 enzyme was located in a self-transmissible IncX3 plasmid and conferred a high level of resistance. Furthermore, the strain harbored multiple virulence genes including *mrkD*, *fimH*, aerobactin, and *rmpA*. The virulence of the strain was evaluated *in vitro* and *in vivo* and showed high virulence in both assays, further suggesting the concomitant presence of both virulence and resistance genes without compromising fitness costs in the strain studied.

Generally*, K. pneumoniae* strains carry only one carbapenemase gene. However, several *K. pneumoniae* strains have been reported to carry two or even three carbapenemase genes (Table 1; Supplementary Table S1). Of note, one study estimated the basal expression levels of *K. pneumoniae* resistance and concluded that the expression levels of β-lactamase and carbapenemase genes were higher in MDR *K. pneumoniae* strains when compared to MDR-hvKp strains, suggesting that the latter have a higher metabolic load due to the multiple resistance and virulence factors (Fursova et al., 2022). Indeed, until very recently, the “relatively low” number of cases of MDR-hvKp may have been due to the high fitness cost of harboring both resistance and virulence traits in the same strain, with the acquisition of resistance genes generally being detrimental to the virulence capabilities of pathogens (Gonzalez-Ferrer et al., 2021). However, as demonstrated in several studies above, several carbapenem-resistant hvKp strains have emerged in recent years, most of which show no compromising fitness cost and with great potential for dissemination. Importantly, a study conducted in China reported a fatal infection of an ST86-K2 NDM-1 and KPC-2-producing *K. pneumoniae* harboring the *iroN*, *iucABCD*, *iutA,* and *rmpA2* genes encoded in a IncHI1/IncFIB plasmid (Liu et al., 2019). Furthermore, the *bla*_NDM-1_ gene was carried in a IncN plasmid, while *bla*_KPC-2_ was carried in a IncFII(K) plasmid. The mouse lethality test results showed that the strain was as virulent as the ST23-K1 serotype strain and that the *bla*_KPC-2_ harboring plasmid was successfully transferred to an *E. coli* J53 recipient strain, highlighting the great potential for virulence and dissemination of this strain.

A study conducted in Italy reported three *K. pneumoniae* strains co-harboring two carbapenemase genes (*bla*_OXA-48_ and *bla*_NDM-1_/ *bla*_NDM-5_) and one *K. pneumoniae* strain co-harboring three carbapenemase genes (*bla*_OXA-48_, *bla*_NDM-1_, *bla*_NDM-5_), while concomitantly carrying key virulence genes (Lorenzin et al., 2022). The isolate co-harboring 3 carbapenemase genes carried an IncFIB/IncHI1B hybrid plasmid with the *bla*_NDM-1_, *iucABCD*, *iutA,* and *rmpA* genes. A similar IncFIB/IncHI1B hybrid plasmid was found in a *K. pneumoniae* isolate in Egypt, carrying the *iucABCD*, *iutA,* and *rmpA* and *rmpA2* genes (Ahmed et al., 2021).

An unusual ST464-K53 co-harboring NDM-1and KPC-2 was reported in China (Hao et al., 2022), showing that the combination of multiple carbapenemase genes in the same strain does not occur only in common sequence types. Furthermore, the strain also harbored *fimABCDEFGHIK*, *mrkABCDFHIJ*, *iroE,* and *entABCDEFS.* Although it did not show a positive string test and did not harbor *rmpA*/*rmpA2* or *aerobactin* genes, the strain showed high virulence in the serum-killing assay as well as in the *G. mellonella* infection model. Another study conducted in China reported an ST11-K64 *K. pneumoniae* strain producing NDM-5 and KPC-2 harbored in a IncFII-type plasmid and IncB/O/K/Z type plasmid, respectively. This strain also carried the *fimABCDEFGHIK*, *mrkABCDFHIJ*, *ybtAEPQSTUX*, *iroEN*, *entABCDEFS*, *iucABCD*, *iutA,* and *rmpA2* genes (Hu et al., 2021).

Several years ago, ESBL (SHV- and TEM-types) encoding genes were observed on virulence plasmids, with the acquisition of these plasmids by the bacteria increasing their virulence potential. Since then, the epidemiology has changed due to the spread of carbapenemase resistance in *K. pneumoniae*. However, until recently, the carriage of plasmids harboring these enzymes did not appear to impose a fitness cost on the bacteria, as the strains were usually less virulent (Hennequin and Robin, 2016). Currently, evidence of the concomitant presence of both virulence and resistance genes within the same strain is increasing dramatically worldwide.

Overall, carbapenem-resistant highly virulent or hypervirulent *K. pneumoniae* did not show compromised fitness and have great potential for dissemination. More pathogenicity and virulence assays, preferably in murine infection models (Russo and Macdonald, 2020), as well as through serum killing assays, are needed to evaluate the overall pathogenicity and virulence of the strain and to help distinguish virulent from hypervirulent strains. Furthermore, it is known that the horizontal transfer of plasmids is often more restricted in strains carrying multiple plasmids than in strains carrying only a single plasmid and that several factors may play a role in the horizontal transfer capacity of multiple plasmids carried by the same host. Nevertheless, more conjugation experiments and plasmid stability assays are needed to better address the potential dissemination of carbapenem-resistant highly virulent or hypervirulent *K. pneumoniae* strains and, together with the virulence assays, to improve the understanding of the overall fitness cost of these pathogens.

## 8 Distribution of virulence genes according to carbapenemase produced in carbapenem-resistant highly virulent or hypervirulent *Klebsiella pneumoniae*

In this review, the average number of virulence genes per strain was superior in the OXA-producing strain (5.8), followed by KPC-producing (4.7), NDM-producing (4.6), IMP-producing (4.4), and VIM-producing strains (2.7). Additionally, strains harboring two or three carbapenemase genes had an average number of 4.2 genes per strain (Supplementary material).

KPC-producing strains were by far the most common and were predominantly found in China. Furthermore, we found that among these strains, *rmpA2*, aerobactin, salmochelin, and *rmpA* encoding genes were the most frequent virulence genes found, while the remaining virulence factors: yersiniabactin, enterobactin, type 1 and type 3 fimbriae, and *peg-344* gene were less frequently found. In addition, among KPC-producing strains in Asia, *rmpA*, *rmpA2*, aerobactin, salmochelin were the most predominant virulence genes found, while in the Americas, type 1 and 3 fimbriae, enterobactin and yersiniabactin were more prevalent (Table 1; Supplementary Table S1).

NDM-producing strains were widespread in Asia, Europe, and Africa showing different virulence distribution. Overall, among these strains, *rmpA*, yersiniabactin and aerobactin were the most abundant genes, followed by type 3 fimbriae, *rmpA2*, salmochelin, enterobactin and type 1 fimbriae. Moreover, *peg-344* was present in only one NDM-producing strain in the studies reviewed (Table 1, Supplementary Table S1).

IMP-producing strains carried 4 equally frequent genes: *rmpA2*, *rmpA*, aerobactin, and salmochelin, followed by the yersiniabactin gene and were reported from Asia. VIM-producing strains were also found in Asia, with *rmpA* and aerobactin being the most frequently identified virulence genes, followed by type 3 fimbriae, yersiniabactin, salmochelin, and *rmpA2*. No type 1 fimbriae, enterobactin, or *peg-344* genes were observed (Table 1; Supplementary Table S1).

OXA producers were reported from Asia and Europe. Yersiniabactin, *rmpA*, salmochelin, aerobactin, *rmpA2* and type 3 fimbriae were the most common genes in OXA-producing *K. pneumoniae* strains in both continents, while type 1 fimbriae, enterobactin, and *peg-344* genes were less common (Table 1; Supplementary Table S1).

**Figure 3:**
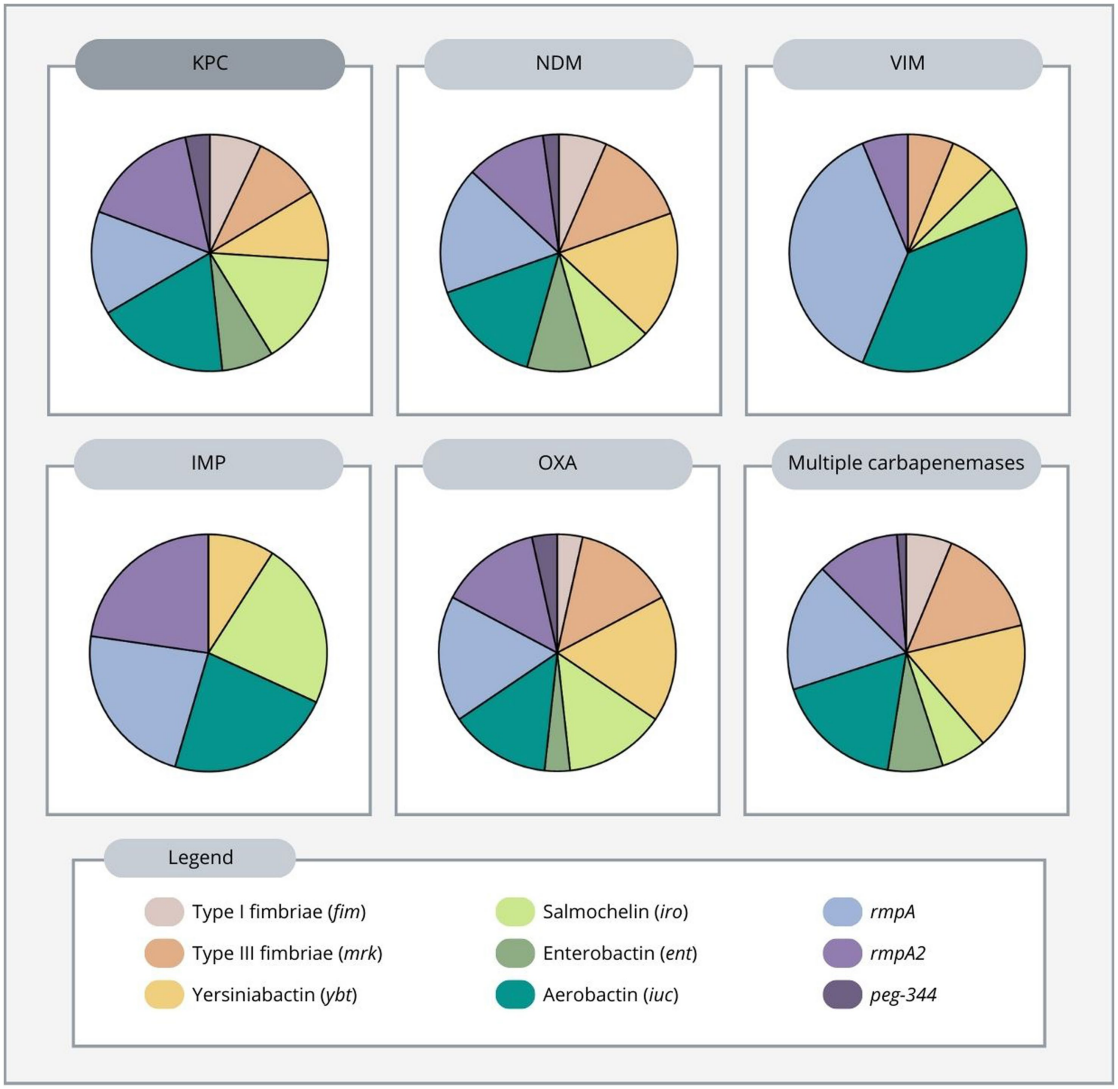
Distribution of virulence genes according to carbapenemase produced in carbapenem-resistant highly virulent or hypervirulent *Klebsiella pneumoniae*.

Multiple carbapenemase-producing *K. pneumoniae* strains have been reported from Africa, Europe and Asia. Aerobactin, *rmpA*, yersiniabactin, type 3 fimbriae and *rmpA2* were the most frequently produced virulence genes, followed by enterobactin, type 1 fimbriae, salmochelin, and *peg-344* gene (Figure 3; Supplementary Figure S1). More specifically, in Africa, the *rmpA*, *rmpA2*, aerobactin and yersiniabactin genes were more common. In Europe, type 3 fimbriae and *rmpA* were more frequent, while in Asia, aerobactin and yersiniabactin were more prevalent, showing a different pattern between continents (Table 1; Supplementary Table S1). Overall, the most frequently produced virulence genes in carbapenem-resistant highly virulent or hypervirulent *K. pneumoniae* strains were aerobactin, *rmpA* and *rmpA2* genes (Supplementary material).

## 9 Distribution of virulence genes in different sequence types in carbapenem-resistant highly virulent or hypervirulent *Klebsiella pneumoniae*

Most highly virulent or hypervirulent carbapenem-resistant *K. pneumoniae* strains belonged to ST11 and ST23, with the former being almost exclusively reported from Asia (mainly from China) and the latter being mainly from Asia and Europe, but also from America (Table 1; Supplementary Table S1). Importantly, ST11 strains reported from China accounted for more than a quarter of all strains collected in this review (Supplementary material). The number of virulence genes also differs between these two high-risk clones, with ST11 having an average of 4.6 virulence genes and ST23 having an average of 4.4. In addition, ST11 strains reported from the African continent showed an average of 6 virulence genes per strain, while ST11 strains reported from the Asian continent showed an average of 4.5 virulence genes. In the American continent, ST11 was found in only one strain carrying 4 virulence genes. ST23 reported from both Europe and Asia had an average of 4.4 virulence genes, while ST23 in America was found in only one strain carrying 5 virulence genes (Supplementary material).

**Figure 4:**
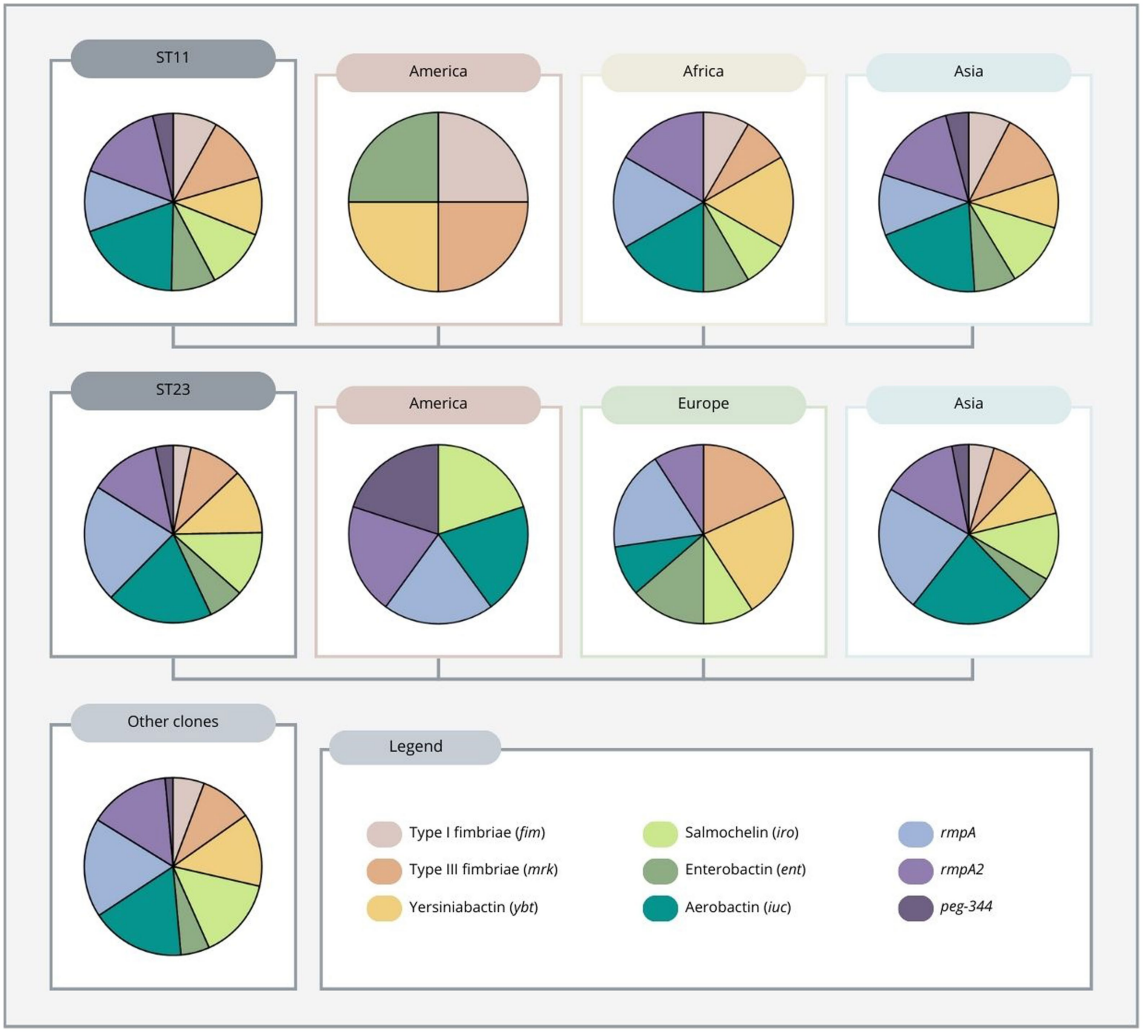
Distribution of virulence genes among ST11, ST23 and other clones (ST13, 14, 15, 16, 29, 35, 36, 65, 86, 101, 147, 258, 268, 375, 383, 392, 395, 420, 437, 464, 592, 859, 893, 1,265, 1,399, 1,660, 2,193 and 2,230) of carbapenem-resistant highly virulent or hypervirulent *Klebsiella pneumoniae*. ST11 and ST23 clones are also differentiated by region.

Regarding ST11 strains reported from the American continent, the only report showed the presence of type 1 and 3 fimbriae, yersiniabactin, and enterobactin. In Africa, yersiniabactin, aerobactin, *rmpA* and *rmpA2* were the most common virulence genes. In Asia, aerobactin and *rmpA2*, followed by type 3 fimbriae, salmochelin, *rmpA* and yersiniabactin, were the most predominant virulence genes found. Overall, aerobactin and *rmpA2* were the most frequently found genes (Figure 4).

These results are worrisome and worth highlighting because ST11 is mainly associated with classical *K. pneumoniae* strains. The susceptibility of CG23 strains to carbapenems and the low virulence of CG258 have allowed most *K. pneumoniae* infections to remain treatable to some extent, but the evolutionary convergence of strains belonging to high-risk and highly disseminated clones such as ST11 (CG258) with simultaneous hypervirulence, multidrug resistance and easy transmissibility of plasmids, represents a new and dangerous threat to public health. Indeed, the convergence of carbapenem resistance and hypervirulence in ST11 clones has been increasingly reported in the last few years (Table 1; Supplementary Table S1), especially since the fatal outbreak in a Chinese hospital in 2016 (Gu et al., 2018).

Furthermore, the majority of ST11 carbapenem-resistant highly virulent or hypervirulent *K. pneumoniae* strains found in the literature were mainly associated with the K64 and K47 loci (Table 1; Supplementary Table S1). The ST11-K64 lineage was reported mostly from China, with one report from Brazil, while the ST11-K47 lineage was reported mainly from China, but also Taiwan, and Egypt. Among the ST11-K64 lineage, aerobactin and *rmpA2* were the most frequent virulence genes found, while ST11-K47 had mostly aerobactin, *rmpA2* and type 3 fimbriae. Of interest, one study conducted in China showed that ST11-KL64 might have emerged from an ST11-KL47–like ancestor through recombination, showing enhanced virulence and transmissibility when compared to ST11-K47 (Zhou et al., 2021).

The ST23 high-risk clone has historically possessed a genetic background conferring hypervirulence and fitness, is mostly associated with the K1 serotype (Bialek-Davenet et al., 2014), and is mainly found in Asian countries (Lee et al., 2017). Although largely susceptible to antibiotics, ST23 carbapenem-resistant highly virulent or hypervirulent *K. pneumoniae* strains have recently been increasingly reported (Table 1; Supplementary Table S1).

Of particular interest is the ECDC (European Center for Disease Prevention and Control) rapid risk assessment report of 2021 on the emergence of hypervirulent high-risk ST23 clones harboring carbapenemase genes in several European countries (European Centre for Disease Prevention and Control, 2021). This report highlights the high dissemination capacity of ST23 clones from Asia to other continents not only in hypervirulent *K. pneumoniae* but also in carbapenem-resistant highly virulent or hypervirulent *K. pneumoniae* strains. Furthermore, this report also emphasizes the need for further epidemiological surveillance and better assessment of virulence markers associated with hypervirulence, particularly through whole-genome sequencing (WGS) analysis.

Regarding the distribution of virulence genes in ST23 reported from the American continent, the only ST23 strain reported carried salmochelin, aerobactin, *rmpA*, *rmpA2,* and *peg-344*. In Europe, yersiniabactin, type III fimbriae and *rmpA* were the most predominant genes found. ST23 strains reported from Asia showed a high prevalence of aerobactin and *rmpA*. (Figure 4). Overall, among all ST23 strains found, *rmpA* and aerobactin were more frequently identified. Additionally, except for an isolate from Russia harboring the K57 serotype (Fursova et al., 2022), all ST23 strains found were associated with the K1 serotype (Table 1; Supplementary Table S1).

Although most of the strains found in this study belonged to ST11 and ST23, other clones were identified, namely ST13, 14, 15, 16, 29, 35, 36, 65, 86, 101, 147, 258, 268, 375, 383, 392, 395, 420, 437, 464, 592, 859, 893, 1,265, 1,399, 1,660, 2,193 and 2,230 (for detailed distribution of virulence factors among these clones, see Supplementary Figure S2). Overall, these clones mainly carried *rmpA*, aerobactin, *rmpA2*, salmochelin, and yersiniabactin (Figure 4). Interestingly, ST258, a dominant clone among carbapenem-resistant *K. pneumoniae* strains in the Americas and southern Europe (Wyres et al., 2020), was not frequently identified in this study, showing that this clade was not frequently reported to harbor both carbapenem-resistant and virulence genes. One of the reasons for its low reporting could be the lack of detection of virulence genes in the European region, as mentioned above. Also, most of the studies collected in this review were from the Asian continent, a region where ST258 is not predominant, in contrast to ST11, a clade from which ST258 originated (Wyres et al., 2020).

Furthermore, it should be emphasized that the predominant capsular types found in hypervirulent strains are K1 and K2, followed to a lesser extent by K5, K20, K54, and K57 (Russo and Marr, 2019). K1 was mainly found in ST23 strains and K2 in ST86 and ST65 (Table 1; Supplementary Table S1). Apart from three studies where a K57 capsular locus was found (Shao et al., 2021; Fursova et al., 2022; Zhao et al., 2022), one study from China reporting a K54 locus strain (Yuan et al., 2019), and three studies reporting a K20 locus (Chen et al., 2020; Shen et al., 2020; Le et al., 2022), no other reports of K57, K54, K20 or K5 carbapenem-resistant highly virulent or hypervirulent *K. pneumoniae* strains were identified. Combined, K1 and K2 strains accounted for 43.1% of all strains collected in this review (Supplementary material), which is considerably lower than the estimated 70% reported in the literature for hypervirulent strains (Russo and Marr, 2019).

Overall, carbapenem-resistant highly virulent or hypervirulent *K. pneumoniae* strains were found more frequently in specific *K. pneumoniae* lineages, namely ST11-K47/K64 and ST23-K1, distributed in different countries. ST11-K47/K64 strains were found mainly in China and to a lesser extent in Brazil and Egypt, while ST23-K1 was mainly found in Asian countries, especially China and Iran, but also in European countries (Spain, United Kingdom, France) and in the United States of America. ST11 strains were more associated with aerobactin and *rmpA2* and ST23 with *rmpA* and aerobactin, although the predominance of virulence genes for both clones varies between different continents. Furthermore, although ST11 strains had a slightly higher average number of virulence genes compared to ST23, ST11 also had a lower dissemination capacity, being reported almost exclusively in China. In fact, overall, 28.6% of the studies included in this review reporting ST23 strains were studies conducted outside Asia, compared with only 13.6% of studies reporting ST11 strains (Supplementary material). Altogether, these results emphasize the great epidemic success of ST11 carbapenem-resistant highly virulent or hypervirulent strains in Asia, a region where hypervirulent strains were mostly associated with ST23 clones. This study also highlights the high dissemination capacity of ST23 among carbapenem-resistant highly virulent or hypervirulent strains, a clone previous associated with infections caused by hvKp strains in Pacific Rim countries (Russo and Marr, 2019).

## 10 Treatment for carbapenem-resistant highly virulent or hypervirulent *Klebsiella pneumoniae*

Most of the current treatment plans for carbapenem-resistant hvKp are the same as the treatment guidelines for carbapenem-resistant *K. pneumoniae*. While one study reported a rapid resistance evolution among the hypervirulent ST11-KL64 clone during treatment with tigecycline and polymyxin (Jin et al., 2021), another study suggested that ceftazidime-avibactam might be a reasonable choice in the treatment of carbapenem-resistant hvKp isolates, particularly for the KPC-2-producing ST11 hvKp isolates (Yu et al., 2018). However, since KPC-producing strains conferring resistance to ceftazidime-avibactam have been emerging worldwide (Mendes et al., 2023), other options such as cefiderocol should be considered. As a novel siderophore cephalosporin designed to treat carbapenem-resistant bacteria, cefiderocol is a suitable alternative for patients infected with strains that are carbapenemase producers, including those that produce metallo-β-lactamases (Bassetti et al., 2021; Kaye et al., 2023). The combination of ceftazidime-avibactam plus aztreonam could also be a suitable option, particularly among metallo-β-lactamases producers (Falcone et al., 2021; Taha et al., 2023). However, it’s important to note that the choice of antibiotics and treatment regimens may vary and should be guided by the phenotypic and genotypic profile of the strain, the patient’s condition, and any underlying health issues.

## 11 Conclusion

Historically, *K. pneumoniae* has been characterized by two very different pathotypes: drug-resistant and hypervirulent. Although they spread independently and distinctly, both pathotypes achieved their epidemic success. While drug-resistant strains were particularly well adapted to healthcare settings, infecting mostly immunocompromised patients, hypervirulent strains were more associated with community-acquired infections in otherwise healthy individuals. Currently, approximately 40 years after the first reports of hvKp strains, we are witnessing the convergence of both pathotypes in the same strain, posing an unprecedented threat to public health. In this study, we focused on the virulence genes produced by these drug-resistant and hvKp strains, particularly in carbapenem-resistant highly virulent or hypervirulent *K. pneumoniae*. Overall, the most frequently identified virulence gene were aerobactin, *rmpA* and *rmpA2*. Most carbapenem-resistant highly virulent or hypervirulent *K. pneumoniae* reported in the literature are KPC-2-producing strains carrying mainly *rmpA2*, aerobactin, salmochelin, and *rmpA*. These pathogens are very often identified with either the high-risk clones ST11 or ST23, the former being linked to K47 or K64 loci and the latter mainly to the K1 serotype. ST11 clones have been reported mainly in Asia with aerobactin and *rmpA2* being the most commonly identified virulence genes, whereas ST23 clones have been reported mainly from Asia, but also from Europe, typically carrying aerobactin and *rmpA* for the former and yersiniabactin and type 3 fimbriae for the latter. Although mostly confined to these two high-risk clones, carbapenem-resistant highly virulent or hypervirulent *K. pneumoniae* strains have also been reported in several other clones, further emphasizing the importance of epidemiologic surveillance for these pathogens.

While some speculations regarding the fitness cost of *K. pneumoniae* harboring both carbapenem resistance and virulence genes in the same strain may still exist, the concomitant presence of these traits is possible with apparently no compromising fitness. These pathogens typically carry one (mainly KPC-2) or even two or three carbapenemase genes, while carrying multiple virulence genes and maintaining a high pathogenicity and overall virulence in several assays, including in both *G. mellonella* and murine infection models. Although the number of reports of carbapenem-resistant highly virulent or hypervirulent *K. pneumoniae* strains is not particularly high (except for the Asian continent, accounting for nearly 80% of the studies included in this review), the emerging number of hospital outbreaks is alarming. Still, we are likely assisting an underreport of these pathogens in several countries, since the detection of virulence genes, as part of diagnostic microbiology routines, is not normally conducted. Therefore, adequate prevention and control measures as well as national WGS coverage with systematic analysis of virulence genes, resistance genes, and epidemiology of these pathogens, are necessary and urgently needed, especially as we observe a shift of infections in healthcare settings where carbapenem-resistant highly or hypervirulent *K. pneumoniae* strains are starting to replace classical *K. pneumoniae* strains.

## Author’s note

### Search strategy and selection criteria

Articles for this review were identified using PubMed. The key terms used were “*Klebsiella pneumoniae*” AND “carbapenem resistance” AND “virulence” or “hypervirulence.” References from relevant articles using the search terms: ‘*Klebsiella pneumoniae*’ AND ‘carbapenem resistance’ or ‘carbapenem-resistant’ AND/OR ‘hypervirulence’ or ‘hypervirulent’ were also collected. Articles search was restricted to English, Spanish, and Portuguese language. Articles from 2017 until October 2022 were considered. All articles that did not present simultaneous information regarding the carbapenemase and virulence genes produced, as well as the clone and capsular loci identification, were not considered.

## Author contributions

GM: Conceptualization, Investigation, Validation, Writing – original draft, Writing – review & editing. MS: Investigation, Validation, Writing – review & editing. JR: Investigation, Writing – review & editing, Validation. AD: Validation, Writing – review & editing. CC: Conceptualization, Supervision, Validation, Writing – review & editing.

## Footnotes

^1^https://www.ncbi.nlm.nih.gov/genbank/
