# Supplementary material for: Virulence factors in carbapenem-resistant hypervirulent Klebsiella pneumoniae
Source: Front Microbiol. 2023 Nov 30;14:1325077. doi: 10.3389/fmicb.2023.1325077 (PMC10720631; doi:10.3389/fmicb.2023.1325077)

Supplementary Material

**Virulence factors in carbapenem-resistant hypervirulent *Klebsiella pneumoniae***

**Gabriel Mendes^1^, Maria Leonor Santos^1^, João F. Ramalho^1^, Aida Duarte^2,3^, Cátia Caneiras^1,3,4*^**

^1^Microbiology Research Laboratory on Environmental Health (EnviHealthMicro Lab), Institute of Environmental Health (ISAMB), Associate Laboratory TERRA, Faculty of Medicine, Universidade de Lisboa, 1649-028 Lisbon, Portugal

^2^Faculty of Pharmacy, Universidade de Lisboa, 1649-033 Lisbon, Portugal

^3^Egas Moniz Center for Interdisciplinary Research (CiiEM), Egas Moniz School of Health and Science, Monte da Caparica, Portugal

^4^Institute of Preventive Medicine and Public Health, Faculty of Medicine, Universidade de Lisboa, 1649-028 Lisbon, Portugal

*** Correspondence:**Cátia Caneiras
ccaneiras@medicina.ulisboa.pt

**Supplementary Figure 1.** Distribution of virulence genes according to carbapenemase produced in carbapenem-resistant highly virulent or hypervirulent *Klebsiella pneumoniae*.


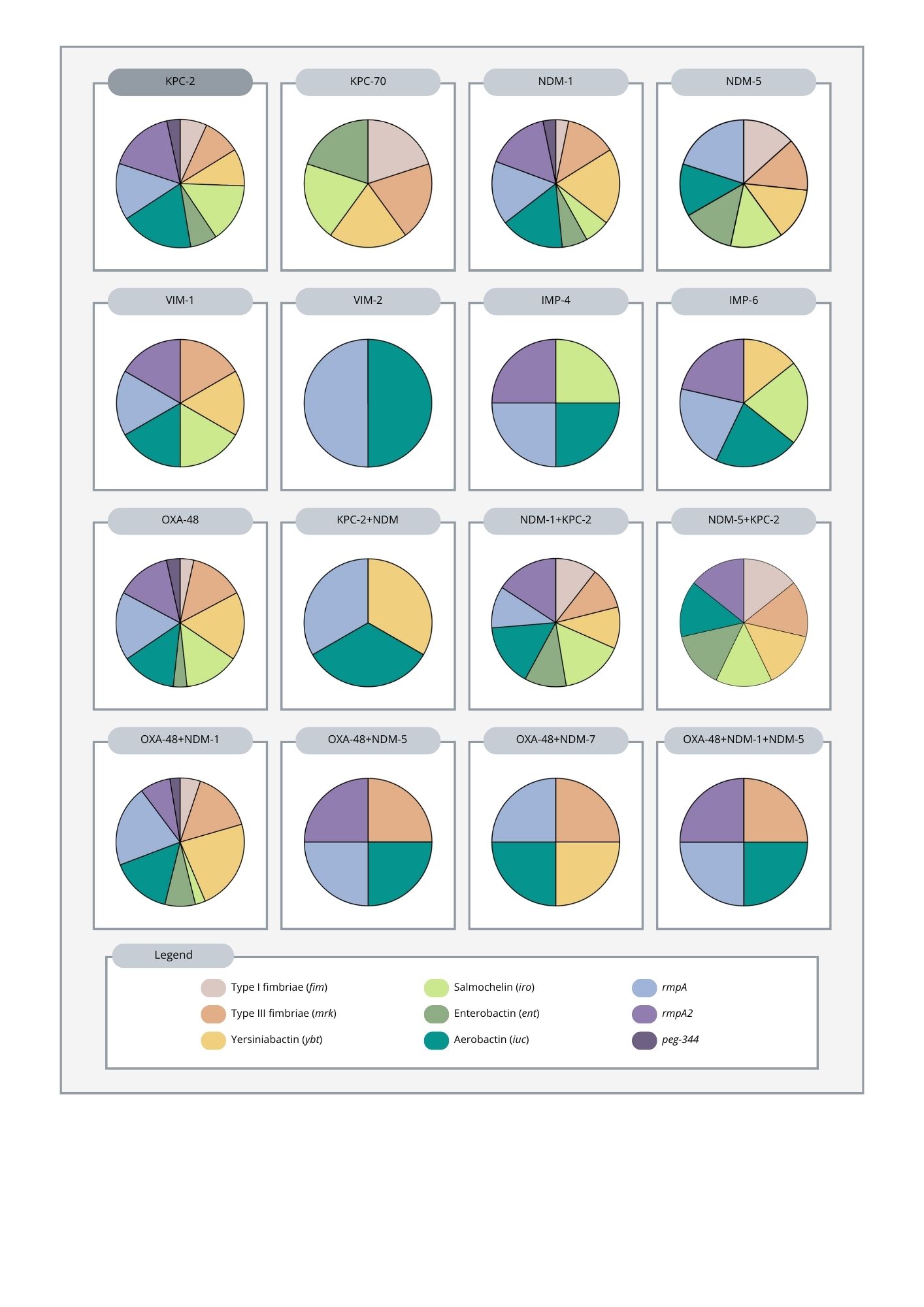


**Supplementary Figure 2.** Distribution of virulence genes among different clones in carbapenem-resistant highly virulent or hypervirulent *Klebsiella pneumoniae*.


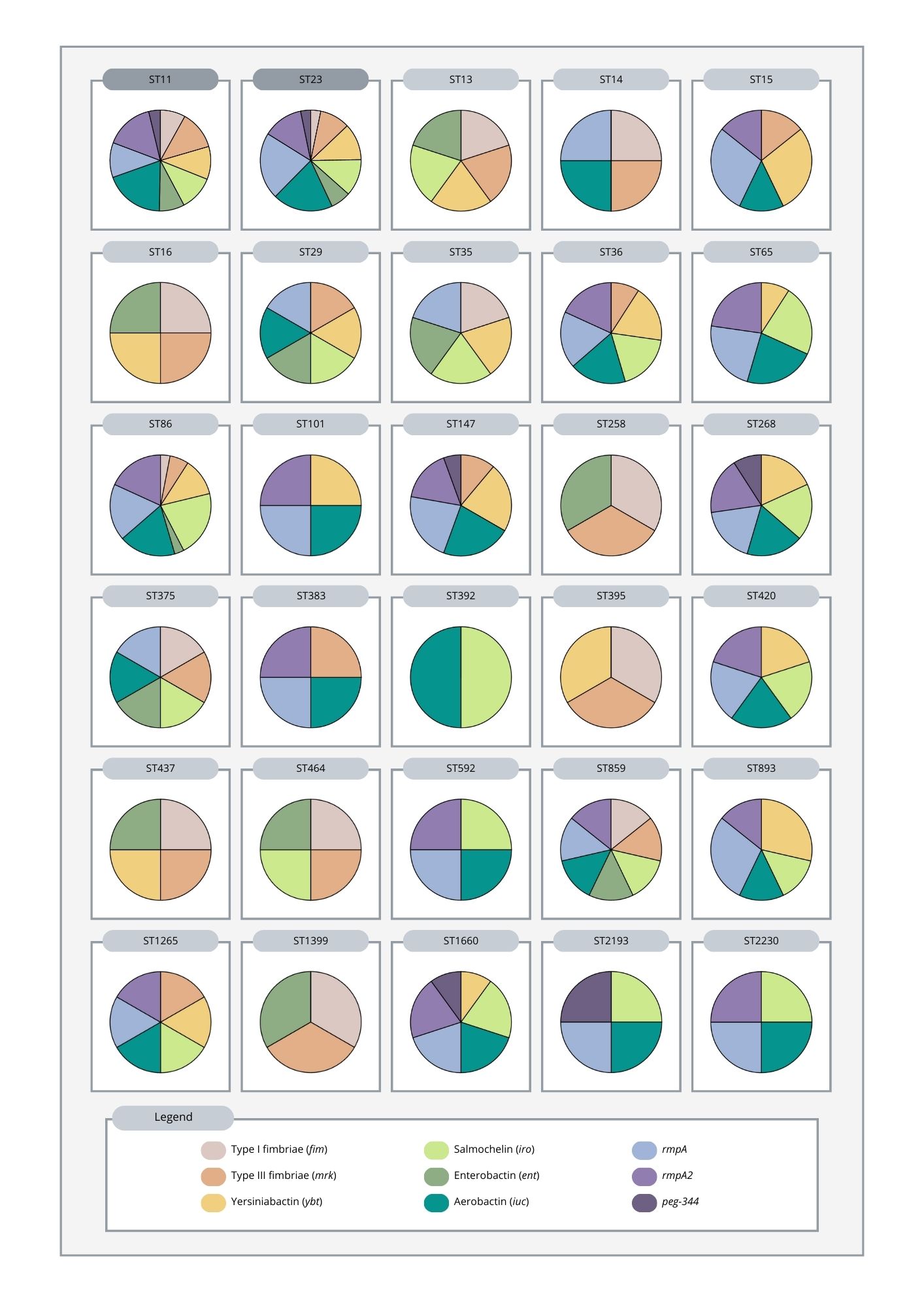

Supplement: Supplementary file 2 [file Data_Sheet_2.docx]
